# Data Standards ≠ Data Quality

**Published:** 2013

**Authors:** Meredith Nahm, W. Ed Hammond

**Affiliations:** Duke Center for Health Informatics, Duke University, Durham, NC, USA

**Keywords:** Data standards, data quality, information quality, Clinical informatics, Clinical research informatics

## Abstract

The relationship between data quality and data standards has not been clearly articulated. While some directly state that data standards increase data quality, others claim the opposite. Depending on the type of data standard and the aspects of data quality considered, both arguments may in fact be correct. We deconstruct a typology of data standards and apply a dimensional definition of data quality to clearly articulate the relationship between the two, providing a framework for data quality planning.

## Introduction

Depending on the type of data standard and the aspects of data quality considered, data standards may or may not impact data quality. Unfortunately, the language we use lacks specificity both about data standards and data quality. Quality likewise is an imprecise term. There are at least two world-views from which quality is approached: 1) quality as conformance to specifications vs. 2) quality as fitness for use. [1] While these need not be different, in practice, there may be divergence. In the absence of context dependent definition of data quality, significant expectation differences between suppliers and customers can result. Likewise, there are different perspectives from which data quality is approached. For example, some view data quality as those things pertaining to the data values themselves while others also include contextual features such as timeliness, and ease of access. Lack of specificity about data standards and data quality enables diverging expectations of the value of data standards and impedes their adoption and progress. We hope instead that clarity in language and intent will hasten the promise of standardization that has benefitted so many other industries.

## Types of Data Standards

We have yet to see a classification of data standards. Such a classification might be based on the aspects of reality (or data about it) that are represented. Such a classification system would surely increase the explicitness with which we talk about data standards, and would aid in clarifying the relationship between data standards and data quality.

## Types of Data Quality

Data quality has been defined as a multidimensional concept. [2] Some dimensions such as accuracy are properties of individual data values and are context independent while others are dependent on the context of use. Data quality dimensions are used to define and articulate expectations of data. Data quality dimensions have been articulated for regulatory decision-making in clinical trials for marketed therapeutics. [3] Such dimensions have not yet been articulated for other healthcare contexts. It is clear, however, that use of data by those other than those who originally collected them, necessitates additional dimensions. Until definitions of data quality for healthcare have been articulated, the term, “data quality” with respect to health care data has little meaning.

## Data Standards and Data Quality

By merely deconstructing 1) the types of data standards and 2) the dimensions of data quality, the inequality of data standards and data quality becomes apparent. Different data uses, necessitate different quality dimensions. Different types of standards impact data quality in different dimensions. With this approach, we have a way to examine a data standard and assess its impact on data quality.

## Supplementary Material

poster
