# Supplementary material for: Data Standards ≠ Data Quality
Source: Stud Health Technol Inform. Author manuscript; Available in PMC 2015 Feb 4. (PMC4317361)
Supplement: poster [file NIHMS659184-supplement-poster.pdf]

Data Standards ≠ Data Quality

Duke Translational Medicine Institute

Meredith Nahm and W. Ed Hammond

Duke Center for Health Informatics, Duke University, Durham, NC, USA

Abstract

The relationship between data quality and data standards has not been clearly articulated. While some informaticians directly state that data standards increase data quality, others describe situations where this is not the case. Depending on the type of data standard and the aspects of data quality considered, both arguments may in fact be correct. We apply a dimensional definition of data quality to clearly articulate the relationship between data standards and data quality, and provide a framework for data quality planning.

Introduction

Unfortunately, the language we use to talk about data quality and data standards lacks specificity. We posit that each impacts data quality differently. Quality likewise is an imprecise term. There are at least two world views from which quality is approached: 1) quality as conformance to specifications (Crosby) vs. 2) quality as fitness for use (Deming, Juran).[1] While these need not be different, in the absence of a clear definition of what quality means for a particular deliverable, significant expectation differences between suppliers and customers can result. Lastly, there are different perspectives of what data quality actually is.

We explore each of these in depth and use the explicit treatment of each to provide a framework describing the relationship between data standards and data quality.

Types of Data Standards

We have yet to see a classification or typology of data standards—a classification based on what aspects of the data are standardized. Such a classification system would surely increase the explicitness with which we talk about data standards. For the purposes of clarifying the relationship between data standards and data quality, we offer the following list.

Data elements

Data elements are the atomic unit of information exchange and use.

Data element sets

A standard data element set is a collection of data elements whose member data elements have been specified. A dataset is a group of data values for such a set of data elements.

Terminologies

A terminology is a set of labels and sometimes numerical codes.

Data or information model

A data or information model is a graphical depiction that can be used to show the following: 1) things about which data are kept, e.g., people, places, ideas, events; 2) relationships between those things; and 3) characteristics (called attributes) of those things.[2]

Ontology

An ontology represents the relationships between universals (and particulars or instances). Ontologies represent the world as it exists rather than the relationships between data that we may collect about the world.

Data exchange models

Data exchange models standardize the syntax, format, mechanism, and expected interactions of transporting data. These models often abstract above the specifics of data elements; thus, while the format is specified, often the specific content is not.

Types of Data Quality

Data quality has been defined by the information quality community as a multidimensional concept.[3] Some dimensions such as accuracy are properties of individual data values and are context independent, while others, e.g., relevance, are dependent on the context of use. Data quality dimensions are used to define and articulate expectations of data, and fit with both "fitness for use" and "conformance to specification" perspectives of quality. When necessary and sufficient dimensions have been articulated and paired with acceptance criteria, the gap between the "fitness for use" and "conformance to specification" perspectives diminishes.

Data quality dimensions have been articulated for regulatory decision-making in clinical trials for marketed therapeutics: Accurate, Legible, Contemporaneous, Original, and Attributable, referred to as the ALCOA criterion.[4] Such dimensions have not yet been articulated for health care contexts. However, that use of data by someone other than whoever collected them, i.e., secondary use, necessitates additional dimensions such as unambiguous definition, as well as context of data collection.

Until necessary and sufficient definitions of data quality for health care have been articulated, the term "data quality" with respect to health care data has little meaning. We posit that for clinical decision-making, five attributes are important: 1) to have accuracy within some wide acceptance criteria; 2) to come from a trusted origin; 3) to be reliably assessed/documented for similar patients in routine care; 4) to have consistent meaning across health care; and 5) to be immediately accessible. If these are the important dimensions, then in a case where the data were accurate, trusted, and reliable but not accessible, a clinician could certainly say the data were of low quality. Unfortunately, standard data elements specifying definition and measurement method may impact dimensions 1 and 3, but probably not 2 and 4. Different types of standards impact data quality in different ways/dimensions.

Data Standards and Data Quality

By deconstructing 1) the types of data standards according to the aspects of data or reality represented and 2) the dimensions of data quality, the inequality of data standards and data quality becomes apparent. With this approach, we can examine a data standard and understand and assess its impact on data quality.

Case 1

A quick examination of the data in the Admission Discharge Transfer (ADT) messages at our institution reveals strings on the following:

999999999
999-9999
NO PHONE
.
N/A
919
000-0000
(555)555-5555
(919)555-1212

Registration information is used in many ways; one is the ADT message that among other things is used to notify relevant parts of the facility of a new patient. The patient identification (PID) section of the standard contains a home phone number attribute (phone number—home, data type XTN [extended telecommunication number]); it is not required, and the standard doesn't enforce other constraints. For data exchange and notification within the facility, the data were of fine quality. For other uses like patient contact, use in entity resolution, billing, and administration, the data were of poor quality, and had a high inaccuracy rate. The ADT message is one of the most relied upon and adopted standards in health care—thus it has had a large positive impact on health care. The ADT message, however, was not developed to address data accuracy, only content and format for exchange; therefore, any expectation that the ADT standard would improve the accuracy dimension of data quality would be misguided.

Case 2

In the recent Institute of Medicine meeting on Sharing Clinical Research Data, Dr. Seyfert-Margolis reviewed a case study in which data from 101 legacy New Drug Applications (NDAs) were converted to the Clinical Data Interchange Standards Consortium (CDISC) Submission Data Tabulation Model (SDTM).[5] She described a situation in which conversion to the SDTM standard resulted in reduction of the information content. She presented an example of multi-racial patients and said, "We saw a lot of interesting stuff around racial categorization." The CDISC SDTM standard follows the Office of Management and Budget (OMB) five-category race and two-category ethnicity standard. Dr. Seyfert-Margolis described mapping of data from mixed-race individuals: The person would be coded as one or the other, and the remaining information would be placed in another column in "an additional information field which became quite complicated when we started looking through it." She emphasized that "even though it was standardized … it didn't ensure quality because those categories were not capturing the actual racial identity of the patients," and she made the point that conversion to a standard has the potential to adversely impact data quality and analysis. She further emphasized that in the case of mapping data to a standard for an intended data use, the scientific questions of the intended use drive the details of the conversion.

The CDISC SDTM standard is an information model with an associated standard terminology. Therapeutic area-specific data elements are not defined; thus, especially in the more normalized SDTM domains, the semantics are underspecified. So, while the standard most definitely improves consistent formatting of data for regulatory submission, it has no mechanism to affect accuracy and, through information reduction, may in fact decrease the ability of the reported data to accurately reflect the actual state of the patient at the stated or implied time point.

It is only through considering the aspects standardized and the dimensions of data quality of import that we are able to clearly articulate what the standard will and will not impact.

Case 3

The third case is reported by Williams et al. in the area of performance measure quality reporting to the Joint Commission (then JCAHO). The purpose of the study was to assess in a stratified sample of 30 accredited hospitals the reliability of indicators in myocardial infarction, congestive heart failure, surgical infections, and pregnancy and related conditions as they were implemented across 3400 US hospitals. The study addressed two questions: 1) how reliably the data elements were collected and reported by hospital medical record abstractors, and 2) the impact of data element reliability on the reliability of the indicators. In the study, Joint Commission staff re-abstracted the 61 indicator data elements for randomly identified cases, and adjudicated discrepancy rates were measured for all data elements utilized in the performance indicators. The data elements for the Joint Commission indicators, accompanied in the technical specifications by definitions and medical record abstraction guidelines covering most major eventualities found in medical record data, are among the most specifically defined that we have seen.[6] Williams et al. reported that the weighted average agreement rate was 91.9% (range 62.4%–100%) with nine data elements having agreement rates below 85%. The mean difference in indicator rates based on the data elements was 4.88% (range 0%–13.3%). They identified discrepancies that had an impact on the interpretation of the indicators. Even in this context with very specifically defined data elements, use of a standard, even when directly targeted at the reliability dimension, fell short; quality control of the subjective abstraction process was required to achieve the desired reliability.

DCRI Communications • August 2013

References

1"Quality." Quality Glossary, American Society for Quality (ASQ). Accessed December 10, 2012, from http://asq.org/glossary/q.html.

2National Institute of Standards and Technology. Integration Definition for Information Modeling (IDEF1X), Federal Information Processing Standards Publication 184. Available from www.itl.nist.gov/fipspubs/idef1x.doc.

3Wang R and Strong D. Beyond accuracy: what data quality means to data consumers. J Manage Inf Syst 1996: 12: 30.

4Department of Health and Human Services, Food and Drug Administration. Guidance for Industry: Computer Systems Used in Clinical Trials. April 1999.

5Seyfert-Margolis V. Cost-benefit analysis of retrospective vs. prospective data standardization. In: Institute of Medicine, Sharing Clinical Research Data. Available from: http://www.iom.edu.

6Williams SC, Watt A, Schmaltz SP, Koss RG, and Loeb JM. Assessing the reliability of standardized performance indicators. Int J Qual Health Care 2006: 18: 246-55.

Acknowledgment

This work was supported by National Library of Medicine Grant 1K99LM011128-01A1. The contents of this poster are solely the responsibility of the authors and do not necessarily represent the official views of the National Institutes of Health.
